# Prevalence and heritability of distichiasis in the English Cocker spaniel

**DOI:** 10.1186/s40575-015-0024-7

**Published:** 2015-08-02

**Authors:** Tanja Petersen, Helle Friis Proschowsky, Tommy Hardon, Søren Nyhuus Rasch, Merete Fredholm

**Affiliations:** Animal Genetics, Department of Veterinary Clinical and Animal Science, Faculty of Health and Medical Sciences, University of Copenhagen, Copenhagen, Denmark; The Danish Kennel Club, Solrød Strand, Denmark; Haslev Dyreklinik, Haslev, Denmark; Nyborg Dyrehospital, Nyborg, Denmark

**Keywords:** Distichiasis, Canine, Cocker spaniel, Genetics, Inheritance, Prevalence, Heredity

## Abstract

**Background:**

Canine distichiasis is a well-known cause of ocular irritation and excessive lacrimation (secretion of tears) in the dog. The term distichiasis originates from the Greek words *di* and *stichos* meaning two and rows, respectively, and as the name implies, the condition is characterized by an additional row of cilia, which erupts on the eyelid margin. Many purebred dogs are known to be predisposed to the condition, with many affected individuals within the populations. Even though the problem is widespread, the exact mode of inheritance and the heredity has not been studied extensively. However, some degree of genetic influence has been assumed, due to the high incidences within specific breeds. In the present study we have examined a cohort of English Cocker spaniels in Denmark to determine the prevalence and heritability of the disease.

**Results:**

Data from English Cocker spaniels with an ECVO eye examination registered between 2004–2013 were included in the study. The number of dogs examined during this period was 799, and the prevalence of distichiasis within this cohort was estimated at 49.31 % with a gender predisposition that females are more likely to get distichiasis than males. The correlation between the distichiasis status of the parents and their offspring revealed a significant association between the breeding combination of the parents and the occurrence of distichiasis in the offspring (*p* <0.0001). A relative risk (RR) ranging from 1.3 to 1.8 demonstrates that offspring of two affected parents are more likely to be affected than offspring descending from either one or two unaffected parents. The heritability was estimated to be moderate to high, i.e., 0.22 to 0.51.

**Conclusions:**

The prevalence of distichiasis in English Cocker spaniels from Denmark, examined in 2004–2013 was shown to be extremely high. The relative risk of developing the disease was 1.3 and 1.8 for offspring of one or two affected parents respectively. This together with the moderate to high heritability of the condition indicates that selective breeding could be used to reduce the incidence of distichiasis.

## Lay summary

Distichiasis has long been recognized as a condition that causes severe discomfort and pain in many species of animals including dogs and humans [15, 22]. Distichiasis is characterized by aberrant lashes on the eyelid margin, from where the eye lashes may impinge on the cornea. Forty years ago, canine distichiasis had a low prevalence in the general dog population (1:133) [19], and it was suggested that a few breeds might be more commonly affected than others [5, 15, 19]. At present, it is well known that distichiasis affects many purebred dogs and occasionally some cross bred dogs as well, and the list of supposedly predisposed breeds now includes 109 different breeds [21]. English Cocker spaniels are known to be predisposed to many different conditions, including several eye diseases [1, 2, 8]. The most prevalent eye diseases reported are distichiasis, cataract and retinal dysplasia [27].

In an attempt to reduce the occurrence of inherited eye diseases, ophthalmic examination in the Cocker spaniel has been recommended since the 1990s, and is now compulsory for dogs intended for breeding within the Danish Kennel Club (DKC) [23]. In 2013 the Danish board of the European College of Veterinary Ophthalmologists (ECVO) introduced a new scheme in order to reduce the incidence of distichiasis in American and English Cocker spaniels [16]. The scheme will run for a trial period of five years, and includes grading distichiasis into three different grades according to the severity of the condition. Spaniels with severe distichiasis are excluded from breeding, and breeders are recommended to breed dogs with moderate distichiasis to unaffected or mildly affected dogs [16].

To date, there are no reported explanations of how, or why, the condition has become so widespread within some breeds. One suggestion, however, may be the increased use of affected dogs for breeding, or perhaps the selection of specific types of Cocker spaniels attending exhibitions, i.e., dogs with long and dense lashes. In this study, we have discovered that dogs diagnosed with distichiasis were used in 70.3 % of all breeding combinations over the last ten years. The most frequently used breeding combination was one affected and one unaffected dog (41.7 %), while using two affected dogs occurred in 28.6 % of matings. Another factor that could be contributing to the high incidence, is the way the sires are used for breeding within a specific population. Many sires, both affected and unaffected, produce far more offspring than the recommended limit of 105 puppies, which makes it quite difficult to measure the effect of preventive actions, and to control inherited diseases, such as distichiasis, within a population.

It could be hypothesized that if the presence of long and dense eyelashes is rewarded in the show ring, and if this is related to an increased risk of distichiasis, this could unfortunately lead to a higher prevalence among show dogs compared to the entire population. However, we have no solid evidence that supports this hypothesis.

## Background

Canine distichiasis is characterized by an additional row of lashes in which the adventitious cilia (distichiae) emerge on the free margin of the eyelids through the meibomian gland orifices [10, 19]. Dogs may be uni-or bilaterally affected and distichiae may be found on one or both lids [19, 24]. The distichiae originate from abnormally located hair follicles within the tarsal plate tissue from which the distichiae grows, or in between the meibomian glands emerging through the meibomian gland orifices. Less frequently, the glands of Moll or glands of Zeis are used as the routes of least resistance, but this is rarer [8, 24]. One or multiple distichiae may use the same orifice, and the affected lid may bear only a few distichiae or, on occasion, the entire length of an eyelid may be involved, suggesting the existence of a double row of lashes in the dog as well [6, 19]. Lashes are only present on the upper eyelid in the dog, so the definition of a second row of lashes would be more analogous to distichiasis in man [19].

Many breeds are known to be predisposed to canine distichiasis, with purebred dogs being particularly affected. Occasionally the condition is seen in crossbred dogs as well [19]. The American and English Cocker spaniels are known to be some of the most frequently diagnosed breeds [4, 6, 19]. The condition is generally considered a congenital hereditary disease [7], but some authors suggest that the condition could be acquired due to long term chronic inflammation of the eyelids and conjunctiva [4, 15]. The condition is usually detected in young and adolescent dogs, with clinical signs manifested from two to six months of age, but the aberrant distichiae may sometimes arise later in life [4, 25, 28]. Acquired distichiasis may be more common in man, where it occurs as a result of ophthalmic diseases, or physical or chemical trauma to the adnexa [3].

It has been suggested that distichiasis is inherited as an autosomal dominant trait, however, this has not been formally proven. Therefore, the exact mode of inheritance and etiology has not been clarified [6, 15]. Nevertheless, due to the high incidence within specific breeds, some degree of genetic influence can be assumed, and the condition is considered a presumed inherited eye disease (PIED) [11, 18]. In a recent study on the systematic and environmental influences and the additive genetic variation of PIED in Tibetan terriers, distichiasis was demonstrated to be a hereditary condition with heritability of 0.043 [11]. Clinical signs associated with distichiasis vary greatly among affected dogs including the size, rigidity and the number of distichiae, although the severity of manifestation is not directly proportional to the number of cilia present [7]. Even though aberrant cilia in contact with the corneal surface are assumed to cause corneal irritation, distichiasis in the dog may be clinically insignificant in some individuals [8, 19]. Corneal irritation leads to trigeminal activation causing excessive tear production (lacrimation), mild conjunctivitis and blepharospasm, which are the most common signs associated with the condition, presented as epiphora and squinting of the eyes [7, 9, 15]. In the more advanced cases, clinical signs are more pronounced, especially when the cornea has been subjected to trauma. Symptoms include photophobia, swelling and hyperemia of the nictating membrane, keratitis and corneal ulceration [15, 19]. The diagnosis is made by identifying one or multiple cilia emerging from the meibomian gland orifices [19], and the treatment of choice depends largely on the severity of the clinical signs. In general, treatment is only considered necessary in clinically affected individuals and may involve either palliative treatment or curative surgery [8, 20]. None of the methods available today are completely satisfactory, and recurrence of disease is likely [8, 10].

In the present study we have examined a cohort of English Cocker spaniels in Denmark to determine the prevalence and heritability of distichiasis, and to evaluate the possibilities of using selective breeding as a means to reduce the incidence of the disease in the population.

## Results

### Animals

Data from 799 English Cocker spaniels with a pedigree registered in the Danish Kennel Club (DKC) together with an ECVO eye examination registered between 2004–2013, were used for the prevalence study. A subset of these dogs (*n* = 549) was included in the study of inheritance and heritability. The requirement for being included in this subset was that both the dog itself and both parents should have a registered ECVO eye examination certificate. In Denmark it is mandatory to chip mark and register all dogs in the Danish Dog Registry. The DKC registered Cocker spaniels constitute approximately one third of all Cocker spaniels in Denmark and thus, the dogs included in this study can be assumed to be representative for the Danish Cocker spaniel population.

### Prevalence and sex distribution

Of the 799 dogs included, 394 dogs were diagnosed with distichiasis, and 405 dogs were unaffected. Thus, the prevalence of distichiasis in the population included in the study is 49.31 %. Twenty-five of the dogs diagnosed with distichiasis had additional ophthalmic diagnoses (6.4 %), whereas 32 of the dogs unaffected with distichiasis, had other ophthalmic diseases (7.8 %). There was a significant gender predisposition within the breed (*χ*^2^ –test 6.7599, *p* = 0.009323), as 121 males and 273 bitches were diagnosed with distichiasis, while 161 males and 244 bitches were unaffected.

### Inheritance and relative risk

As shown in Table 1, the 549 dogs for which the distichiasis status was registered in both parents were distributed in the following mating combinations: 157 dogs (28.6 %) produced by two affected parents; 229 dogs (41.7 %) produced by one affected and one unaffected parent (111 (48.5 %) and 118 dogs (51.5 %) of the 229 dogs with an affected father and mother respectively); and 163 dogs (29.7 %) produced by two unaffected parents. A significant association between the breeding combination of the parents and the prevalence of distichiasis in the offspring (*p* <0.0001) was detected. As shown in Table 2, the occurrence of distichiasis in the offspring was not significantly different when comparing the breeding combinations unaffected x not affected and not affected x not affected. However, a highly significant difference was found in the occurrence of distichiasis in the offspring descending from two affected parents compared to offspring descending from either one or two unaffected parents. The relative risk (RR) that the offspring will be affected by distichiasis increases proportionally with the number of affected parents used in the breeding combination. If one of the parents is affected, the relative risk is 1.3 times higher compared to using two unaffected dogs. If two affected dogs are used, the relative risk is 1.4 times higher compared to using one affected and one unaffected dog and 1.8 times higher compared to using two unaffected dogs (Table 2). However, both affected offspring from two unaffected parents, and unaffected offspring from two affected dogs were observed. These data, together with the increasing relative risk of disease in offspring with one and two affected parents respectively, indicate that distichiasis can be characterized as a threshold character. Threshold characters are traits that vary in a discontinuous manner, but are not inherited in a simple Mendelian manner.

**Table 1:** The association of the distichiasis status of the parents and their offspring

Breeding combinations	Offspring with distichiasis	Offspring without distichiasis	Total number
Affected x affected (+/+)	105	52	157
Affected x not affected (+/−)	106	123	229
Not affected x not affected (−/−)	60	103	163
Total	271	278	549

**Table 2:** Statistical significance of association

Compared breeding combinations	*X* ^2-test^	*p*-value	95 % CI	RR
+/− vs. −/−	3.13	0.08	[−0.01; 0.20]	1.3
+/+ vs. +/−	15.11	<1.0 x 10^−4^	[0.10; 0.31]	1.4
+/+ vs. −/−	27.76	<1.4 x 10^−7^	[0.19; 0.41]	1.8

+Parent affected by distichiasis. -Parent not affected by distichiasis

### Heritability

The scale of liability (standard deviation from threshold) within the population of dogs comprising offspring from unaffected parents, and offspring from one and two affected parents, respectively, were used for the estimation of heritability according to published methods described for threshold characters [14]. The heritability (h^2^) of distichiasis was estimated at 0.22 when using offspring from one affected parent and 0.51 when using offspring from two affected parents.

### Sires

In order to limit the detrimental effects of popular sires, the Danish Kennel Club has defined recommended maximum limits for the number of offspring per sire in each breed. The limits are defined as 25 % of the average yearly number of registrations as proposed by Indrebø (2008) [17]. For the English cocker spaniel, this limit corresponds to 105 offspring [26]. A total of 150 sires fathered the subset of 549 dogs included in the inheritance study. Within the 150 sires, 55 were diagnosed with distichiasis, while 95 were unaffected by the condition. Six of the 55 sires with distichiasis (11 %) and four of the 95 sires without distichiasis (4 %) exceeded the recommended limitation. The number of exceeded offspring ranged from 5 to 69 puppies (mean 35.4). Over the ten year period 2004–2013, English Cocker spaniel sires without distichiasis were preferentially used for breeding. In total, sires without distichiasis produced 3820 puppies (mean 40.2) distributed in 756 litters. Sires diagnosed with distichiasis produced 2708 puppies (mean 49.2) distributed in 509 litters.

### The grading scheme

The grading of distichiasis in mild, moderate or severe was introduced on 1st August 2013. Therefore, only 135 of the 390 dogs diagnosed with distichiasis had a grade in addition to being registered as affected with distichiasis (36.4 %). The 135 dogs that had been graded included 116 (86 %) mild, 15 (11 %) moderate and four (3 %) severe.

## Discussion

Canine distichiasis is considered to be a presumed inherited eye disease (PIED) in dogs, with the American and English Cocker spaniels being some of the most frequently diagnosed breeds [4, 6, 19]. The prevalence found in this study (49.31 %) is considerably higher than that of earlier studies performed in this breed. The highest prevalence found in earlier investigations was 26 %, including both English and American cocker spaniels [4, 6, 19], which is about half of what was detected here. The population included in this study represents approximately one third of the Danish Cocker spaniel population and thus, can be assumed to be fairly representative for the entire population. There is, however, a possible bias in our data because the registration of eye results is a requirement for obtaining studbooks for any offspring. Thus many of the dogs registered with eye examinations results in the DKC database, are likely to have been tested because the owner wants to use the dog for breeding.

Many of the dogs included in this study are presumably intended for breeding, because they have been judged, and awarded prizes at dog shows before the eye examinations. It could be hypothesized that if the presence of long and dense eyelashes is rewarded in the show ring, and if this is related to an increased risk of distichiasis, this could unfortunately lead to a higher prevalence among show dogs compared to the entire population. However, we have no solid evidence that supports this hypothesis.

We found a significant association (*p* < 0.0001) between the breeding combination of the parents and the prevalence of distichiasis in the offspring. The relative risk (RR) of producing affected offspring was found to increase with the number of affected parents in the breeding combination. I.e., in offspring produced by one affected and one unaffected parent the relative risk was 1.3 times higher compared to the risk in offspring produced by two unaffected dogs. In offspring produced by two affected dogs the relative risk was 1.4 times higher compared to offspring from the previously mentioned mating combination and 1.8 times higher compared to offspring produced by two unaffected dogs. Hence, the risk of producing dogs that will develop distichiasis at some point in their lives was almost twice as high when mating two affected dogs. These observations together with other data showing that simple Mendelian inheritance is unlikely, lead us to conclude that distichiasis is most probably inherited as a threshold character. Thus, we disagree with earlier assumptions that canine distichiasis is inherited as a dominant trait [6, 15].

The heritability of distichiasis was estimated according to methods described for threshold characters ([14]) and found to be in the range of 0.22-0.51 depending on whether the estimate was based on the offspring from matings between one affected and one healthy parent or matings between two affected parents. The discrepancy between the two estimates might be explained by increased inbreeding within one of the breeding combinations. Heritability for distichiasis has also been estimated in Tibetan terrier [18]. In this breed the heritability was estimated at 0.043, however, since the prevalence in the Tibetan terrier study population was much lower (11.43 % of 849 dogs) and since the heritability was calculated using estimates of additive genetic variation, the heritability estimates in the two populations are not directly comparable.

Although there have been no analyses of changing disease incidence with time, within the population, anecdotally, the proportion of affected animals has been increasing over the last few years. To date, there are no reported explanations of how, or why, the condition has become so widespread within some breeds. One suggestion, however, may be the increased use of affected dogs for breeding, or perhaps the selection of specific types of Cocker spaniels attending exhibitions, i.e., dogs with long and dense lashes. In this study, we have discovered that dogs diagnosed with distichiasis were used in 70.3 % of all breeding combinations over the last ten years. The most frequently used breeding combination was one affected and one unaffected dog (41.7 %), while using two affected dogs occurred in 28.6 % of matings. Another factor that could be contributing to the high incidence, is the way the sires are used for breeding within a specific population. Many sires, both affected and unaffected, produce far more offspring than the recommended limit of 105 puppies, which makes it quite difficult to measure the effect of preventive actions, and to control inherited diseases, such as distichiasis, within a population.

The grading scheme was introduced six months prior to this study, and since it is relatively new, no publications or statistics are available about distribution of the different levels of disease within the predisposed breeds. In this study, the majority of the graded dogs were only mildly affected, and moderate to severe cases were quite rare. This is consistent with the fact that distichiasis does not always cause clinical signs in the Cocker spaniel, and the identified distichiae are most frequently small and soft [6, 7]. Corneal ulcerations are also infrequently described in the literature [8, 19]. For now, there is no written standardisation for the characterisation of the different grades of disease severity. This may cause inconsistency in the distribution of the results, thus giving a misleading impression of the severity of the condition within a specific breed, as the grading is made solely on the subjective opinion of the eye scheme examiner. If the results are considered consistent, the scheme will be most applicable for identifying the general distribution of the disease within a population. Dogs with severe disease are excluded from breeding [16].

This study is to our knowledge the first study of prevalence and heritability of distichiasis in the Cocker spaniel. Since we have estimated a moderate to high heritability of the condition it will be possible to use selective breeding to reduce the incidence of disease. The high prevalence within this breed makes it impossible to exclude all affected animals without depleting the gene pool. The present breeding recommendations tries to overcome this by excluding only the severely affected. Thus, reducing the incidence of distichiasis must be regarded as a long time breeding goal.

## Conclusion

The presence of breed predispositions and the relatively high occurrence within the population suggests a genetic influence for canine distichiasis. In the Cocker spaniel we have found that the risk of getting affected offspring increases when one or more parents are affected by distichiasis, suggesting the accumulation of presumed predisposing genes. These observations allow us to conclude that distichiasis is inherited as a threshold character. The moderate to high heritability that we have estimated of the condition indicates that selective breeding, primarily using unaffected or mildly affected dogs for breeding, will reduce the incidence of the disease.

## Methods

### Study design

Data from ECVO-certificates issued during 2004–2013 were retrieved from the Danish Kennel Club and analyzed in a retrospective cohort study. A total of 799 cocker spaniels were included and divided into two groups, based on their distichiasis status (distichiasis yes/no). Dogs with a registered ophthalmic examination without an ECVO-certificate were excluded, in order to ascertain that all dogs in the study had been examined in the same manner. Diagnosis of distichiasis was based on all ECVO-examinations performed during the lifespan of the individual dog, and comprised both confirmed diagnoses and comments regarding the presence of one or more distichiae. Several dogs were eye examined more than once during the study period. A dog was regarded as affected if just one of the examinations resulted in a distichiasis diagnosis. Additional status on the severity of the condition (if present) and presence of other ophthalmic conditions were listed. In order to evaluate the mode of inheritance and heritability, 549 of the initial 799 cocker spaniels were selected based on the availability of a valid ECVO-certificate on both of their parents. Dataset on the sires used to produce the selected 549 dogs, included number of litters and puppies produced both in total and on average in the breed. The “limit of number of puppies produced by popular sires” was outlined and the number of sires exceeding this limitation was noted.

### Prevalence and Sex distribution

All of the 799 cocker spaniels with a valid ECVO-certificate issued during 2004–2013 were included to demonstrate the occurrence in the population and the distribution of the condition between sexes. The chi square test was used to test if the distribution between the sexes was significantly different.

### Inheritance and relative risk

The 549 selected dogs were divided into two groups based on their distichiasis status, and further subdivided into three sections based on the breeding combination of their parents. The three breeding combinations of the parents were based on their distichiasis status, and consist of Affected x Affected, Affected x Unaffected and Unaffected x Unaffected. The relative risk was calculated to measure the association between the different levels of exposure and the risk of getting affected offspring.

### Heritability

The scale of liability (standard deviation from threshold) within the population of dogs comprising offspring from unaffected parents and offspring from one and two affected parents respectively were used for the estimation of heritability according to methods described for threshold characters [12]. The calculations were performed using the program available at http://www.ihh.kvl.dk/htm/kc/popgen/genetik/applets/heritt.htm [13].

### The grading scheme

The grading of distichiasis into mild, moderate and severe, has been introduced on an experimental basis, and no official written ECVO guidelines are available. As a rule of thumb; mild cases include a total of one to five cilia in all four palpebrae, and moderate cases includes a total of five to ten cilia in all four palpebrae. Severe cases include more than ten cilia in all four palpebrae, but the clinical health of the eye is also taken into consideration. If changes secondary to distichiasis are present in the eye, the grading will be moderate or severe regardless of the number of cilia.

## Abbreviations

CI: Confidence interval
ECVO: European college of veterinary Ophthalmologists
h^2^: Heritability
p: *p*-value
PIED: Presumed inherited eye diseases
RR: Relative risk
